# Data on synthesis and characterization of new diglycerol based environmentally friendly non-isocyanate poly(hydroxyurethanes)

**DOI:** 10.1016/j.dib.2015.11.034

**Published:** 2015-11-22

**Authors:** Mariusz Tryznowski, Aleksandra Świderska, Zuzanna Żołek-Tryznowska, Tomasz Gołofit, Paweł G. Parzuchowski

**Affiliations:** a Warsaw University of Technology, Faculty of Chemistry, Poland; b Warsaw University of Technology, Faculty of Production Engineering, Poland

## Abstract

This article contains original experimental data, figures and methods to the preparation of non-isocyanate poly(hydroxyurethanes) by an environmentally friendly method without the use of toxic phosgene and isocyanates from bis(2,3-dihydroxypropyl)ether dicarbonate and various diamines (Tryznowski et al., Submitted for publication) [1]. Bis(2,3-dihydroxypropyl)ether dicarbonate was obtained from a one-step procedure from commercially available diglycerol. The product was characterized by ^1^H NMR, ^13^C NMR, and FTIR spectroscopies and for the first time by X-Ray diffraction measurements. Then, the bis(cyclic carbonate) monomer was used as a precursor for the synthesis of various NIPUs. The NIPUs were prepared in a non-solvent process. Spectral and thermal properties of the NIPUs are compered. Here we give the procedure in order to perform bis(2,3-dihydroxypropyl)ether dicarbonate with high yield and the procedure NIPU synthesis and the complete set of monomer and NIPU analysis (^1^H NMR, ^13^C NMR, FTIR, X-Ray).

**Specifications Table**

**Table:** 

Subject area	*Chemistry*
More specific subject area	*Polymer Chemistry and Cyclic carbonate synthesis*
Type of data	*Figure, X-Ray image, Text file*
How data was acquired	*FTIR (Biorad FTS-165 FTIR spectrometer) ^1^H NMR and ^13^C NMR (Varian VXR 400* MHz *spectrometer), X-Ray (Bruker D8 Venture Photon 100 CMOS diffractometer), DSC (TA Instruments Q2000)*
Data format	*Synthesis procedure of monomer (raw), NIPU polymerization procedure (filtered)*
Experimental factors	– *Diglycerol and various diamines were used as received* – *Solvents were dried prior to use*
Experimental features	– *Bis(2,3-dihydroxypropyl)ether dicarbonate was prepared by a reaction of commercially available diglycerol and dimethyl carbonate* – *Synthesis of NIPU were carried out by a solvent-free and catalyst-free method*
Data source location	*Faculty of Chemistry, Warsaw University of Technology, Warsaw, Poland*
Data accessibility	*Data are available with this article*

**Value of the data**

• The data shows one-step procedure of synthesis of five-membered bis(cyclic carbonate)-bis(2,3-dihydroxypropyl)ether dicarbonate from commercially available diglycerol with high yield.
• The data shows the X-Ray structure of the obtained bis(cyclic carbonate).
• The data shows the use of bis(2,3-dihydroxypropyl)ether dicarbonate as a monomer for the preparation of poly(hydroxyurethanes) in a reaction with various diamines by a solvent-free green route.

## 1. Experimental design, materials and methods

### 1.1 Materials

Diglycerol (α,α-diglycerol ~84%, α,β-diglycerol ~14%, β,β-diglycerol <1%, cyclic diglycerols ~0.2%) was a gift from Solvay S.A. (Brussels, Belgium). 1,2-Diaminoethane (99.5%), 1,4-Diaminobutane (99%), 1,6-Diaminohexane (98%), 1,8-Diaminooctane (98%), 1,12-Diaminododecane (98%), 1,8-Diamino-3,6-dioxaoctane (98%), 4,7,10-trioxa-1,13-tridecanediamine (97%), poly(propylene glycol) bis(2-aminopropyl ether) Mn ~230 (total amines 8.1 meq/g), poly(propylene glycol) bis(2-aminopropyl ether) Mn ~400 (total amines 1.64 meq/g), and 1,3-Bis(aminomethyl)benzene (99%) were purchased from Aldrich Chemical (Poznan, Poland) and used as received. Solvents were purchased from POCh (Gliwice, Poland) and dried prior to use.

### 1.2 Instrumentation

Obtained products five-membered bis(cyclic carbonate) and NIPUs were characterized according well known methods as presented in [1].

FTIR spectra were recorded on a Biorad FTS-165 FTIR spectrometer as KBr pellets or an Bruker ALPHA FTIR spectrometer equipped with a Platinum ATR single reflection diamond ATR module. ^1^H NMR and ^13^C NMR spectra were recorded on a Varian VXR 400 MHz spectrometer using tetramethylsilane as an internal standard and deuterated solvents (CDCl_3_, DMSO-d_6_) and analyzed with MestReNova v.6.2.0-7238 (Mestrelab Research S.L) software. The X-ray measurement was performed at 100(2)K on a Bruker D8 Venture Photon 100 CMOS diffractometer equipped with a mirror monochromator and a CuKα INCOATEC IμS micro-focus source (*λ*=1. 54178 Å). The raw frame data were collected using the Bruker APEX2 program [2] while the frames were integrated with the Bruker SAINT software package [3] using a narrow-frame algorithm integration of the data and were corrected for absorption effects using the multi-scan method (SADABS) [4]. The non-hydrogen atoms were refined anisotropically. All hydrogen atoms were placed in their calculated positions and refined within the riding model. The atomic scattering factors were taken from the International Tables [5].

### 1.3 Synthesis of five-membered bis(cyclic carbonate): bis(2,3-dihydroxypropyl)ether dicarbonate

1000 g (6.02 mol) of diglycerol (a mixture of isomers) was placed in a 5 L three neck round bottom flask equipped with a magnetic stirrer, a thermometer and a reflux condenser, followed by 3210 g (35.6 mol, 3 L) of dimethyl carbonate and 5 g (36 mmol) of K_2_CO_3_. The reaction mixture was heated at 70 °C for 24 h. Then 1.8 dm^3^ of the mixture of dimethyl carbonate and methanol was distilled off at atmospheric pressure at 65 °C over a period of 6 h. Then the reaction mixture was cooled down to room temperature. The precipitated catalyst was filtered off and washed with dimethyl carbonate. The combined organic phases were evaporated to dryness and crystallized from ethyl acetate. A total of 876 g (4.01 mol) of 2 was obtained as a white solid with 79% yield (with respect to α,α-diglycerol).

Yield 876 g (79%); mp 66–67 °C (lit. [6] 62–64 °C); density 1.4981±0,022 g/mL. ^1^H NMR (DMSO-d_6_, 400 MHz); *δ* (ppm)=4.98–4.89 (m, 2H, CH_2_CHCH_2_), 4.52 (t, 2H, *J*=8.5 Hz, OCH_2 cycl._), 4.28–4.20 (m, 2H, OCH_2 cycl._), 3.79–3.72 (m, 2H, OCH_2_), 3.71–3.64 (m, 2H, OCH_2_);); ^13^C NMR (DMSO-d_6_, 100 MHz); *δ* (ppm)=154.8 (C=O), 75.4 (CH), 70.4 (CH_2 cycl._), 65.9 (CH_2_); FTIR (KBr): ν (cm^−1^)=2994, 2927, 2886, 1785, 1486, 1372, 1343, 1252, 1177, 1148, 1113, 1057, 959, 855, 770, 714, and 598.

Crystal data: orthorombic, Pbca, *a*=7.6790(8) Å, *b*=11.7916(12) Å, *c*=20.679(2) Å, *α*=90°, *β*=90°, *γ*=90°, *V*=1872.4(3) Å^3^ *Z*=8, *μ*=1.219 mm^−1^, and *D*_calc_=1.548 g/cm^3^, 13,108 reflection measured (4.28≤2*Θ* ≤70.05°), 1783 independent (*R*_(int)_=0.0444). The final *R*_1_ was 0.0396 (*I*>2*σ*(*I*)) and w*R*_2_ was 0.1065 (all data).

**Fig. 1:**
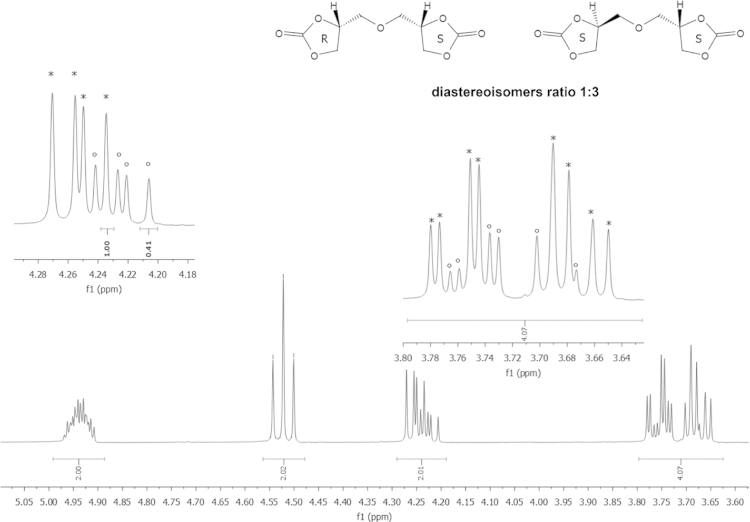
^1^H NMR spectra of bis(2,3-dihydroxypropyl)ether dicarbonate 2 diastereoisomers.

**Fig. 2:**
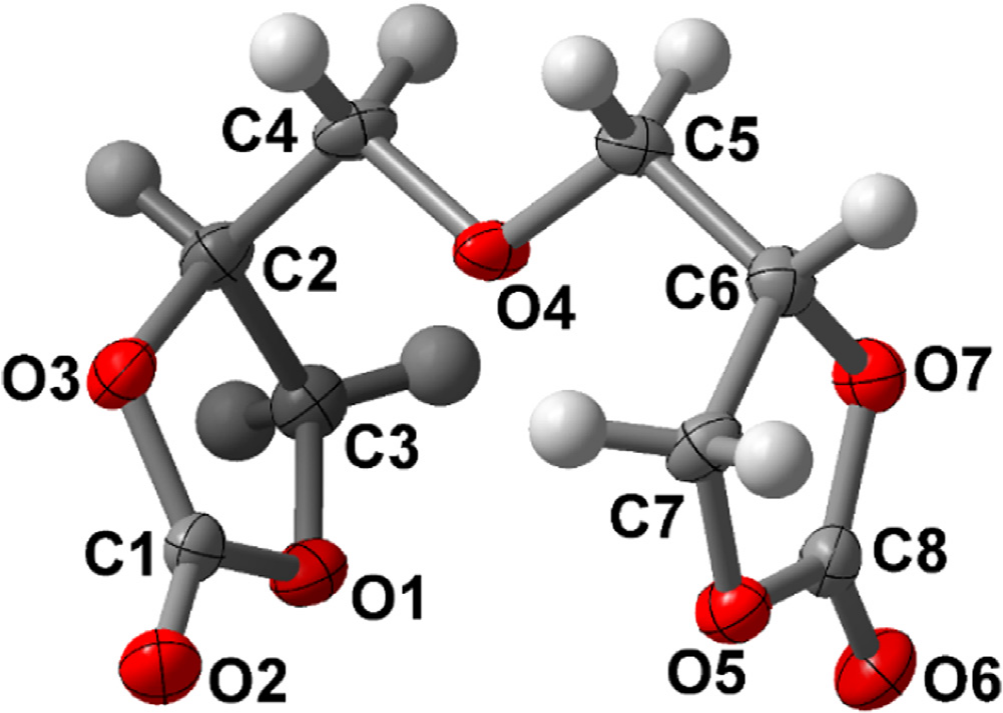
X-Ray structure of bis(2,3-dihydroxypropyl)ether dicarbonate 2.

**Scheme 1:**
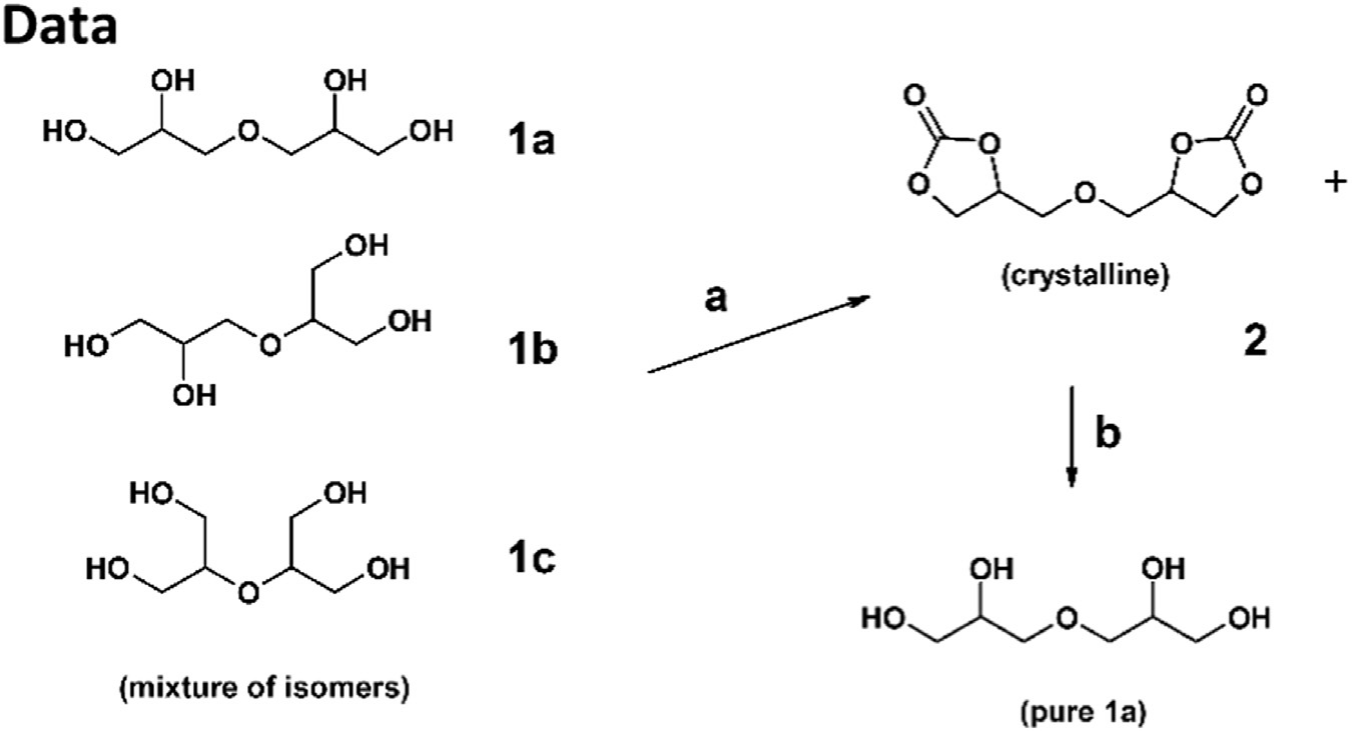
Synthesis and hydrolysis of 2. Reaction conditions: a) DMC, K_2_CO_3_; b) MeOH, H_2_O, K_2_CO_3_.

Bis(2,3-dihydroxypropyl)ether dicarbonate, 2 was prepared by a reaction of commercially available diglycerol (Solvay) and dimethyl carbonate (Scheme 1). The five-membered bis(cyclic carbonate) was obatined with a 79% yield in contrast to the multistep procedure described previously in the literature [6], [7]. The formation of the five-membered cyclic carbonates was confirmed by FTIR spectroscopy (see Fig. 1 in Ref. [1]), ^1^H NMR and ^13^C NMR (Fig. 1). The structure of the product was determined by single-crystal X-Ray diffraction (Fig. 2). The thermal properties of 2 were characterized by DSC and TGA.

### 1.4 Synthesis of NIPU

**Scheme 2:**
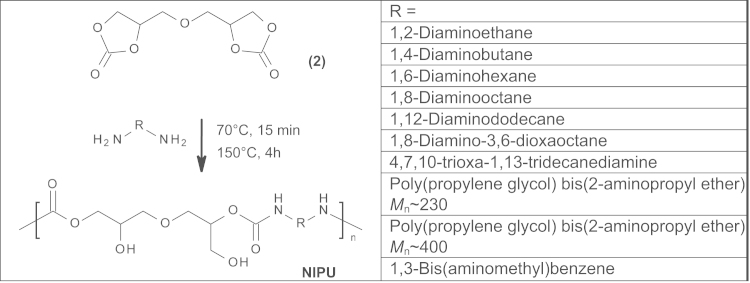
Synthesis of NIPUs from 2 and various diamines.

In a typical run, NIPUs were synthesized by the reaction of five-membered bis(cyclic carbonate) 2 with various diamines (see Scheme 2 or Table 1 in Ref. [1]). 54.5 g (0.25 mol) of bis(cyclic carbonate) was placed in a 250 mL round bottom flask equipped with a mechanical stirrer and a nitrogen inlet. The solid was melted under nitrogen atmosphere at 80 °C. Then 0.25 mol of an appropriate diamine (see Table 1 in Ref. [1]) was added in portions during 30 min and the temperature increased up to 150 °C. The reaction mixture was stirred at this temperature for 8 h. Then the reaction mixture was cooled down and the polymers analyzed without further purification.

**NIPU 1**; Yield 99.5%; ^1^H NMR (DMSO-d_6,_ 400 MHz); *δ* (ppm)=7.08 (bs, 1.82H, NH_(E)_), 6.76 (bs, 0.18H, NH_(Z)_), 4.98 (bs, 1.4H, OH), 4.79 (bs, 0.6H, OH), 4.68 (bs, 0.8H, CHO(CO)N), 3.98–3.78 (m, 2.5H, CH_2_OCH_2_), 3.78–3.66 (m, 1.4H, CHOH), 3.56–3.21 (m, 5.3H, CH_2_O), 3.00 (bs, 4H, CH_2_NH); ^13^C NMR (DMSO-d_6_, 100 MHz); *δ* (ppm)=156.4, 156.0 (C=O), 73.3, 72.6, 72.5, 69.9, 69.8, 67.8, 65.6, and 60.1 (CH_2_O and CHO), 40.2 (CH_2_NH); FTIR (KBr): *ν* (cm^−1^)=3337, 2944, 2878, 1786, 1697, 1533, 1262, 1140, and 776.

**Fig. 3:**
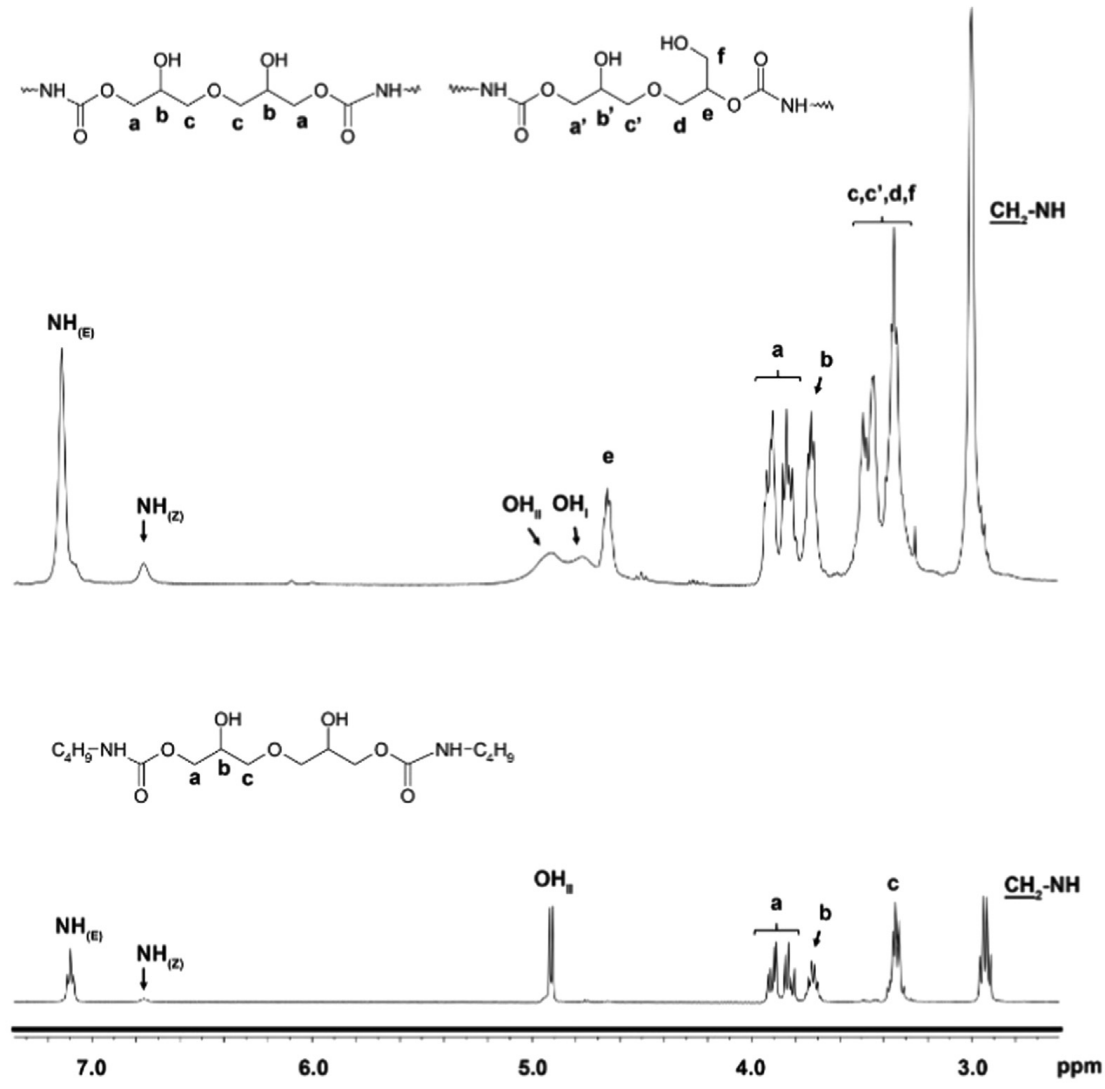
The ^1^H NMR (DMSO-d_6_, 400 MHz) spectrum of the NIPU1 polymer compared to the model compound adduct of bis(cyclic carbonate) with butylamine.

The obtained bis(cyclic carbonate) was used as a monomer for the synthesis of non-isocynate poly(hydroxyurethane)s *via* a polyaddition reaction with various diamines: aliphatic ones, oligoetherdiamines and, for comparison, aromatic diamines (Scheme 2). The polyaddition reactions were carried out by a solvent-free and catalyst-free method. The reaction of bis(cyclic carbonate) with diamines was monitored by IR spectroscopy The formation of NIPU confirmed by ^1^H NMR and ^13^C NMR (Fig. 3). The properties of obtained NIPU 9–NIPU 10 are showed in [1] (Table 2).
